# *Helicobacter pylori* infection, a risk factor for Type 2 diabetes mellitus: a hospital-based cross-sectional study among dyspeptic patients in Douala-Cameroon

**DOI:** 10.1038/s41598-020-69208-3

**Published:** 2020-07-22

**Authors:** Laure Brigitte Kouitcheu Mabeku, Michelle Larissa Noundjeu Ngamga, Hubert Leundji

**Affiliations:** 1 Microbiology and Pharmacology Laboratory, Department of Biochemistry, Faculty of Science, University of Dschang, P. O. Box 67, Dschang, Cameroon; 2 Gastroenterology Department, Laquintinie Hospital of Douala, P. O. Box 4035, Douala, Cameroon

**Keywords:** Diabetes, Stomach diseases

## Abstract

Diabetic mellitus patients are usually prone to chronic infections. However, there have been contradictory reports about the association between *H. pylori* infection and type II diabetes. The present study is aimed at evaluating the prevalence of *Helicobacter pylori* infection among type 2 dyspeptic diabetic patients in the littoral region of Cameroon. This cross sectional study comprised 93 type 2 diabetic dyspeptic patients and 112 non-diabetic dyspeptic patients attending the Gastroenterology Department at two reference hospitals in Douala-Cameroon. The study was approved by the local Ethical Committee of Medical Sciences. Participants were screened for the presence of both type 2 diabetes and *H. pylori* infection. Body mass index (BMI) of all the participants was also recorded. Data was analyzed using SSPS statistical package. *H. pylori* infection was found in 73.11% of diabetic patients versus 58.05% in non-diabetic participants, this difference was found to be significant (OR = 1.472, *p* = 0.0279). This relationship persists even when adjusted to factors such as age and income level of participants. Infected participants from age group ≥ 55 years and those with high income were those with a higher risk to develop diabetes. Infected patients with high BMI were more prone to develops diabetic mellitus compared with infected patients with normal BMI (*p* = 0.0034). Also, participant with high BMI were more prone to develops diabetic mellitus whether they were infected or not. Patients having both *H. pylori* + ve and BMI ≥ 25 kg/m^2^ were significantly more affected by diabetic mellitus than those in the others combined groups (*p* < 0.0001), suggested that high BMI and *H. pylori* infection together or not are factors that favor diabetes mellitus development. Separately or not, *H. pylori* infection and high BMI were risk factor for diabetes mellitus in our milieu.

## Introduction

Diabetes mellitus (DM) is an illness which mainly characterized by a high blood glucose level (hyperglycemia) with defects in carbohydrates, protein and fat metabolism due to either absolute or relative deficiency of insulin and/or action. Regarding statistics from the World Health Organization (WHO), the most common type of diabetes is type 2 diabetes mellitus (DMT2) with about 90% of diabetic patients suffering from this type^1^. This type 2 diabetes mellitus is an evolving pandemic which is responsible for about 3.8 million of adult deaths worldwide^1^, anticipation from the WHO states that the number of adults deaths from diabetes will double by 2030^1^.

Long term damage, dysfunction and failure of various organs are associated with chronic hyperglycemia. Also, susceptibility to certain infections can accompany this chronic hyperglycemia^2–4^. Ricci et al. after investigation reported that upper gastrointestinal tract symptoms similar to those associated with *Helicobacter pylori* (*H. pylori*) infection are more common in patients with diabetes than in individuals with no diabetes (controls)^5^. A report from Hungary suggested that there is an increase in the prevalence of *H. pylori* infection among diabetes mellitus patients^6^ and this report was later supported with studies from developing and developed countries^7–9^. Also, the relationship between *H. pylori* seropositivity, *H. pylori* cagA positivity and higher mean HbA1c levels, an indicator of chronic hyperglycemia has been documented^10^. Another study carrying out on Taiwanese patients revealed that *H. pylori* chronic infection enhance the level of HbA1c and reduces the level of insulin production ^11^. *H. pylori* is a gram negative, microaerophilus bacterium which selectively colonizes the human stomach mucosa. Gastric colonization with *Helicobacter pylori* results in local inflammation in nearly all hosts. This persistent process increases the risk of developing atrophic gastritis, intestinal metaplasia, and noncardia gastric adenocarcinoma^12^. Although many studies have reported coherence of *H. pylori* infection with the DMT2, some have proven poor or no correlation between *H. pylori* infection and DMT2. A study on 195 diabetics has shown a lower seroprevalence of *H. pylori* in type I and II diabetic patients compared with the healthy population^13^. Another study from Xia et al. has shown that *H. pylori* infection is not associated with diabetes mellitus or with upper gastrointestinal symptoms in DM patients^14^. Anastasios et al. in their cross-sectional study of 172 dyspeptic patients (67 diabetics and 105 non-diabetic subjects) found that there was no difference between DM and non-diabetic patients with regard to the prevalence of both *H. pylori* infection and *H. pylori*-related gastroduodenal disorders^15^. Similarly, the report from Jones et al. showed that *H. pylori* infection is not associated with delayed gastric emptying or upper gastrointestinal symptoms in diabetes^16^.

*H. pylori* infection seems to be common in Cameroon: a study carried out in 2004 had demonstrated a prevalence of 92.2% among apparently healthy children in the Buea and Limbe health districts of Cameroon^17^; a hospital-based survey conducted in 2013 has revealed an overall prevalence of 72.5% in Yaoundé, center region of Cameroon^18^. Still in the same region in 2015, a prevalence of 79.3% of this pathogen was documented among children and adolescents with peptic ulcer disease^19^. In 2018, Kouitcheu et al. found a seroprevalence of 64.34% among patients with symptoms of dyspepsia or other symptoms referable to the proximal alimentary tract in the littoral region of Cameroon^20^. There are few data in the literature revealing a broad spectrum of resistance of *H. pylori* clinical isolates circulating in Cameroon against antibiotic currently used in the eradication of this pathogen^21^. Despite the high prevalence of *H. pylori* infection in Cameroon, the broad spectrum of resistance and the high resistance rate of these circulating strains, there is no surveillance programs that monitor the evolution of *H. pylori* resistance in order to allow timely adaptation of the treatment regimens in the country. Regarding diabetes mellitus, epidemiological studies have shown that, in Cameroon 10% of the population is diabetic and that 90% of them are obese at the beginning of the disease^22^. As Sub-Saharan Africa countries, type 2 is the most common type of diabetes in Cameroon. There is lack of information about the prevalence of type 1 diabetes in Cameroon whereas a prevalence rate of 1.5–6.6% of type 2 diabetes was found from 1990 to 2003^23,24^. As far as we know, there is no document reporting the relationship between *Helicobacter pylori* infection and type 2 diabetes in Cameroon. Therefore, the aim of this study was to evaluate the prevalence of *H. pylori* infection in DMT2 dyspeptic patients in Cameroon. For this purpose, data was analyzed from participants attended two public hospitals in Douala metropolis; Laquintinie Hospital and Bonassama District Hospital for dyspepsia. The diabetes status was gotten by the detection of HbA1c blood level. We also found if high BMI, *H. pylori* infection alone or together are associated with diabetes status. A good understanding of the role of *H. pylori* in diabetes mellitus could be useful in terms of prognosis and management of the disease.

## Methods

### Study area

The study was conducted in Douala metropolis, the capital of the Littoral Region of Cameroon with an area covering about 210 square kilometers and an estimated population that surpasses 3,000,000 inhabitants. Douala is located on the banks of the Wouri River. It has an average annual temperature of 27.0 °C, an average humidity of 83% and an average annual rainfall of 3,600 mm^25,26^. It is the commercial and economic capital of Cameroon and the entire CEMAC region. Due to its highly developed infrastructure and peaceful environment for successful business, Douala attracts people from many other parts of the country and from other countries in the region.

### Study population

This study was carried out in two selected health facilities in Douala metropolis; Laquintinie Hospital and Bonassama District Hospital, from August to December 2014. We employed a consecutive sampling for data collection, requesting consent from all dyspeptic volunteer patients who fulfilled the eligibility criteria of the study. All patients aged 35 years and above, either sex, and attending the Gastroenterology Department at the selected health centers for dyspepsia were recruited. We have chosen to include people older than 35 years in order to increase the possibility to have eligible participants, since the risk of developing type 2 diabetes increases with age in developing countries with age ranges from 45 to 64 as the peak age^24,27–29^. Exclusion criteria were (1) Dyspeptic patients less than 35 years; (2) Non-cooperative dyspeptic patients who refused to give their consent or to participate to the study; (3) Dyspeptic patients who received ulcer treatment within the last three months; (4) Dyspeptic patients currently using proton pump inhibitor; (5) Dyspeptic patients with a history of gastric cancer, Dyspeptic patients with a history of *H. pylori* eradication treatment or antibiotics consumption within the last fourth weeks, Dyspeptic diabetic patients under diabetes medication. Pregnant and breastfeeding women were also excluded from the study.

### Variables

The socio-demographic parameters (age, sex), socioeconomic class or income level [low income (≤ 2,500 $/month) taking as a reference, Middle income (2,500–8,500 $/month) and High income (≥ 85 00 $/month)], as well as the following information [history of gastric cancer, previous history of ulcer treatment; previous history of *H. pylori* eradication treatment (triple or quadruple therapeutic regimen) and antibiotics consumption; proton pump inhibitor consumption; diabetes status and history on diabetes medication] were requested from the dyspeptic subjects in a structured questionnaire.

Body mass index **(**BMI) was calculated as weight/height (kg/m^2^) from body weight (Kg) and height (m) of each participant. Participants were classified according to BMI in 2 groups; normal with BMI < 25 kg/m^2^ and overweight or high BMI with BMI ≥ 25 kg/m^2^.

In all volunteer dyspeptic patients who fulfilled the eligibility criteria of the study, the presence of *H. pylori* was investigated by a chromatographic immunoassay for the qualitative detection of antibodies to *H. pylori* and the screen for the absence or presence of type 2 diabetes through blood glycated hemoglobin (HbA1c) level detection. Participants were then divided into two groups; diabetic group, containing dyspeptic patients of type 2 diabetes mellitus with positive or negative *H. pylori* status while non-diabetic or control group, contained non-diabetic dyspeptic individuals with positive or negative *H. pylori* status.

### Specimen collection and processing

Venous blood samples were collected from all subjects. Blood samples (5 ml each) were drawn by a well-trained nurse into vacutainers and plastic tubes. About 2.5 ml blood was placed into ethylene diamine tetra acetic acid (EDTA) vacutainer tubes to perform HbA1c. The remaining quantities of blood (2.5 ml) were placed in tubes without anticoagulant and were left for 10 mn at room temperature to allow blood to clot. Serum samples were obtained by centrifugation at 3,000 rpm for 10 min.

For each serum collected, 3 drops were used to detect *Helicobacter pylori* antibodies using a rapid chromatographic immunoassay commercial kit, DiaSpot^R^ *H. pylori* One Step Test Device (DiaSpot *H. pylori*, Indonesia). This test qualitatively and selectively detect *H. pylori* antibodies in serum or plasma by utilizing a combination of *H. pylori* antigen coated particles and anti-human IgG. This test has sensitivity > 95.9% and specificity about 75.9% with overall accuracy of 85.2% as compared with culture/histology of endoscopic specimens for *H. pylori*.

Blood glycated hemoglobin (HbA1c) levels were used in this study to determine the diabetes status of our sample population. Blood HbA1c level for each patient was determined by ion exchange chromatography method using GLYCOHEMOGLOBIN (SGM, Italia) kit. For this purpose, 100 µl of blood collected in EDTA tubes were used according to the manufacturer’s instruction. The percentage of glycated hemoglobin was determined using a spectrophotometer at 450 nm. The test sensitivity in term of detection limit was 0.0151%. Participants received a positive type 2 diabetes status if glycated hemoglobin level was higher than the cutoff point value recommended by the American Diabetes Association 2012^30^. As recommended, an HbA1C value of 6.5% was considered as the cutoff point in the Diagnosis of Diabetes Mellitus.

### Statistical analysis

Data were expressed either as the mean ± SD and percentage. For continuous variables, comparison between groups was performed using Unpaired t-test and the corresponding *p* value was determined. The chi-square test, Fischer’s exact test were used for categorical variables. A control group constituted of non-diabetic dyspeptic patients was used to compare the strength of the association between *H. pylori* infection and type 2 diabetes mellitus, between BMI status and type 2 diabetes mellitus and between both *H. pylori* infection/BMI status and type 2 diabetes mellitus. The odd ratio (OR) and the corresponding 95% confidence intervals (95% CI) were used to summarize the strength of those associations. Age grou* p* ≥ 55 years old, BMI ≥ 25 kg/m^2^ , and having both *H. pylori* −ve and BMI < 25 kg/m^2^ were choose as reference groups and compare with the other corresponding groups. Significance was accepted at the level of *p* < 0.05. A logistic regression analysis was done for factors with *p* value less than 0.05 or near 0.05 to confirm the impact of this variable on the relationship between *H pylori* infection and diabetes mellitus. Statistical analyses were performed using SPSS Statistics 20.0 software (IBM Corp., Chicago, USA). The primary outcome measure was to identify the relationship between type II diabetes mellitus and *H. pylori* infection among patients with gastroduodenal disorders in Cameroon. Secondary outcome measures included the impact of both *H. pylori* infection and high body mass index on diabetic status and blood HbA1c level of these dyspeptic patients.

### Informed consent for study participation

Each potential participants received an information notice, oral explanation of the study and were clearly informed on the potential risks and benefits of the study and measures taken for confidentiality. Only potential participants who accepted to participate and provided a written consent were enrolled. So, participation was voluntary and each subject involved in the study gave a written consent. The written consent were enrolled of the current study are available from the corresponding author on reasonable request.

### Ethical statement

The study has been performed in accordance with the Declaration of Helsinki of 1975 and its later amendments or comparable ethical standards. The protocol was approved by the local Ethical Committee of Medical Sciences (Approval n°1501/AR/MINSANTE/HLD/CM from Laquintinie Hospital and n°25/AR/MSP/DRSPLT/SSDB/HDB from Bonassama District Hospital).

## Results

### Characteristics of the study population

**Figure 1:**
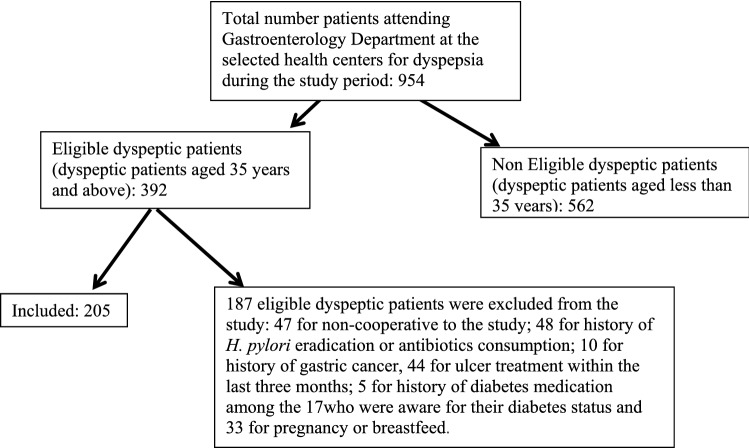
Sketch outlining the selection of our sample population.

Figure 1 illustrates all the dyspeptic patients attending Gastroenterology Department at the selected health centers during the study period and the sample eventually recruited into the study. Just 17 of the eligible subjects (392) were previously aware of their diabetes status and five of them were excluded because of history of diabetes medication.

Two hundred and five subjects with dyspepsia symptoms were enrolled in this study, 78 males and 127 females, giving a sex ratio of 1/1.63. Fifty four percent (53.65%; 110/205) of the subjects were unemployed (low income or poorly skilled class) followed by 26.34% (54/205) from middle class with 2,500–8,500 $/month and 20% (41/205) from elite class with relatively high income (≥ 8,500 $/month).

Participants were divided into 2 main groups according to the diabetic status: Diabetic group; it included 93 type 2 diabetes mellitus dyspeptic patients, 32 males and 61 females. Their ages ranged between 35–75 years, with a mean value of 54.70 ± 1.072 years. Non diabetic group or control group includes 112 non diabetic dyspeptic subjects, 46 males and 66 females with the same age range above and mean age of 53.01 ± 1.104 years (Table 1).

**Table 1:** Socio demographic, socioeconomic, anthropometric parameters and *H. pylori* status according to diabetic status among the dyspeptic patients.

Variable	Diabetic N (%)	Non-diabetic N (%)	[t value] ; {X^2^} OR (95% CI)	*p* value
**Age, Mean ± SD**	**54.70 ± 1.072**	**53.01 ± 1.104**	**[1.086]**	**0.2786**
35–44	17 (18.27)	30 (26.78)	{10.58}	0.0143*
45–54	17(18.27)	32(28.57)		
55–64	45(48.39)	30(26.78)		
≥ 65	14(15.05)	20(17.97)		
Total	93 (45.36)	112 (54.63)		
**Gender, Ratio**	1/1.91	1/1.43		
Female	61 (48.03)	66 (51.96)	1.329 (0.7514–2.349)	0.3864
Male	32(41.02)	46 (58.97)		
Total	93 (45.36)	112 (54.63)		
**Socio-economic class (Income level $/month)**				
Unemployed or low class (≤ 2,500)	49 (44.54)	61 (55.45)	{0.06448}	0.9683
Middle class (2,500–8,500)	25 (46.30)	29 (53.70)		
Elite (≥ 8,500)	19 (45.34)	22 (53.66)		
Total	93 (45.36)	112 (54.63)		
**BMI, Mean ± SD**	**26.25 ± 0.2741**	**24.34 ± 0.2496**	**[5.179]**	< 0.0001*
BMI ≥ 25(kg/m^2)^	72 (77.41)	42 (37.5)	5.714 (3.078–10.61)	< 0.0001*
BMI ˂ 25 (kg/m^2)^	21 (22.58)	70 (62.5)		
Total	93 (45.36)	112 (54.63)		
***H. pylori status***				
Positive	68 (73.11)	65 (58.03)	1.967 (1.087–3.557)	0.0279*
Negative	25 (26.88)	47 (41.96)		
Total	93	112		
**Combination H pylori and BMI status**				
*H pylori* + ve/BMI ≥ 25 kg/m^2^	52 (55.91)	28 (25)	{37.13}	< 0.0001*
*H pylori* –ve/BMI ≥ 25 kg/m^2^	20 (21.50)	13 (11.60)		
*H pylori* + ve/BMI < 25 kg/m^2^	16 (17.20)	37 (33.03)		
*H pylori* –ve/BMI < 25 kg/m^2^	5 (5.37)	34 (30.35)		
Total	93	112		

N: number, Mean ± SD, SD: Standard deviation, (95% CI): 95% confidence intervals, OR: Odd ratio; X^2^: chi-square value in {}, t value in [],*p* value in bold are for t value.

*Significant.

### Distribution of diabetes mellitus status according to sociodemographic, socioeconomic and anthropometric parameters among dyspeptic patients

When examining the distribution of diabetes mellitus as regards sociodemographic and economic factors, we noticed a significant distribution of this pathology with respect to the age of our dyspeptic sample population (X^2^ = 10.58, *p* = 0.0143). The majority of dyspeptic diabetic patients belonged to 55–64 years age group with 58.1 ± 6.01 years as the mean age (Table 1), so this age group was taken as reference group for comparison using univariate and multivariate logistic regression. This positive association between diabetes and age of participant persists even with logistic regression analysis (*p* = 0.0079 and 0.0039) (Table 2).

**Table 2:** Diabetic status adjusted to sociodemographic and economic factors, combined *H. pylori* infection with BMI status of the study population using univariate and multivariate logistic regression analysis.

Variable	N	Diabetic n = 93	Non-diabetic n = 112	Univariate logistic regression		Multivariate logistic regression	
				OR (95% CI)	*p* value	OR (95% CI)	*p* value
**Age ≥ 55 years**							
Yes	96	59 (54.13)	50 (45.87)	0.4647 (0.2679- 0.8676)	0.0079*	0.2714 (0.112–0.6575)	0.0039*
No	109	34 (35.42)	62 (64.58)				
**Gender**							
Female	127	61 (48.03)	66 (51.96)	1.329 (0.7514–2.349)	0.3864	0.5411 (0.2423–1.2087)	0.1342
Male	78	32(41.02)	46 (58.97)				
**Low Income level (2,500 $/month)**							
Yes	110	49 (44.55)	61 (55.45)	0.9311 (0.534–1.623)	0.8882	3.2972 (1.3254–8.2022)	0.0103*
No	95	44 (46.32)	51 (53.68)				
***H. pylori + ve***							
BMI ≥ 25 kg/m^2^	114	52 (45.61)	28 (24.56)	3.6893 (1.7337–7.8506)	0.0007*	3.3289 (1.4898–7.4381)	0.0034*
BMI < 25 kg/m^2^	91	16 (17.58)	37 (40.66)				

N or n: number, BMI: Body mass index, (95% CI): 95% confidence intervals, OR: Odd ratio.

*Significant.

As gender concerned, dyspeptic women were at a slightly higher risk than dyspeptic men to develop diabetes; 48.03 females vs 41.02% males were diabetic (Table 1). But the difference was not significant. This relationship was similar even after multivariate analysis (Table 2).

The rate of diabetes mellitus was almost the same among the different socioeconomic class, although participants with low income were less affected (*p* = 0.9683) (Table 1). But when examining this association using multivariate logistic regression taken low income as reference group, we noticed that participants with low income were significantly with a lower risk of being diabetic. Our data showed that, among the 93 diabetic patients, 44.55% versus 46.32% were with low income, whereas in the non-diabetic group, 55.45% versus 53.68% were with low income. This difference was significant (*p* = 0.0103) (Table 2).

Regarding BMI status, 55.61% (114/205) of this dyspeptic population were overweighed with mean BMI of 26.25 ± 0.2741 and 24.34 ± 0.2496 kg/m^2^ respectively for diabetic and non-diabetic groups. This mean BMI value among diabetic dyspeptic patients was significant high compared to non-diabetic dyspeptic patients (t = 5.179, *p* < 0.0001). Similarly, our results showed a significantly higher prevalence of overweighed dyspeptic patients among diabetic patients compared to non-diabetic ones, 77.41% versus 37.5% respectively (OR = 5.714 (3.078–10.61), *p* < 0.0001) (Table 1).

### *Helicobacter pylori* infection status among dyspeptic diabetics and non-diabetic patients

Among the 205 dyspeptic participants, 133 were *H. pylori* positive, giving an overall seroprevalence of 64.87% in our sample population. Our result showed that diabetic dyspeptic patients were 1.967 time prone to *H. pylori* infection than non-diabetic dyspeptic ones (OR = 1.967, 95% CI: 1.087–3.557). *H. pylori* infection was found in 73.11% (68/93) of the type 2 diabetic patients while it was present in 58.03% (65/112) of the non-diabetic participants. This difference was found to be significant (*p* = 0.0279) (Table 1).

When adjusting *H. pylori* prevalence among diabetics and non-diabetics patients according to the age, socioeconomic and BMI status using logistic regression, the positive relationship between diabetes mellitus and *H. pylori* infection persisted (Table 3).

**Table 3:** impact of *H. pylori* infection on diabetes mellitus adjusted to age, sex, BMI and socio economic status among the study population using univariate and multivariate logistic regression analysis.

Variable	N	Diabetic/*H. pylori* + ve n = 68	Non-diabetic/*H. pylori* + ve n = 65	Univariable logistic regression		Multivariable logistic regression	
				OR (95% CI)	*p* value	OR (95% CI)	*p* value
**Age ≥ 55 years**							
Yes	109	44 (64.70%)	26 (40%)	0.3634 (0.1801–0.7331)	0.0047*	0.2681 (0.1105–0.6505)	0.0036*
No	96	24 (35.29%)	39 (60%)				
**Gender**							
Female	127	40 (31.49)	40 (31.49)	0.8182 (0.4092–1.6362	0.5705	0.5848 (0.2636–1.2974)	0.187
Male	78	28 (35.89)	26 (33.33)				
**BMI ≥ 25 kg/m**^2^							
Yes	114	52 (76.47%)	28 (43.07%)	0.2479 (0.1178–0.5214)	0.0002*	0.2825 (0.1291–0.6184)	0.0016*
No	91	16 (23.52%)	37 (56.92%)				
**Low Income level (2,500 $/month)**							
Yes	110	39 (35.45)	36 (32.72)	1.3119 (0.6636–2.5937)	0.4349	2.9314 (1.1878–7.2345)	0.0196*
No	95	37 (38.94)	21 (22.10)				

N or n : number, + ve: positive, BMI: Body mass index, (95% CI): 95% confidence intervals, OR: Odd ratio.

*Significant.

We noticed that diabetic dyspeptic patients from ≥ 55 years age group were more infected than their corresponding non-diabetic age group. The difference between the two groups was significant (*p* = 0.0036) (Table 3).

Similarly, infected participants with low income were less affected by diabetes than those from the other economic class. The difference between the two groups was significant when using multivariable analysis (*p* = 0.0196) (Table 3).

As regards BMI status, diabetic dyspeptic patients with high BMI were more infected that those with normal body weight. 76.47% versus 43.07% of infected diabetic dyspeptic patients were obese compared to infected non-diabetic dyspeptic patients. The difference was found to be significant (*p* = 0.002 and 0.0016 respectively for uni- and multivariate analysis) (Table 3).

When considering BMI and *H. pylori* status together, we realized that, the rates of patients having both *H. pylori* + ve and BMI ≥ 25 kg/m^2^ were 55.91% in the diabetic patients and 25% in the non-diabetic patients. Also, the rates of patients having both *H. pylori* –ve and BMI < 25 kg/m^2^ were 30.5% in the non-diabetic patients and 5.37% in the diabetic patients (Table 1). So, this later group was taken as a reference group for comparing individual combined category of *H. pylori* and BMI status. As regards *H. pylori* status, we noticed that infected patients with high BMI were 3.3289 fold more prone to develops diabetic mellitus compared to infected patients with normal BMI (OR (95% CI): 3.3289 (1.4898–7.4381), *p* = 0.0034) (Table 2). As BMI status is concern, we noticed that infected patients with high BMI (OR (95% CI):12.63 (4.440–35.92), *p* < 0.0001) and non-infected patients with high BMI (OR (95% CI): 10.46 (3.246–33.72), *p* = 0.001) were significantly more affected by diabetic mellitus compared with patients having both *H. pylori* –ve and BMI < 25 kg/m^2^ (reference group) (Table 4).

**Table 4:** Effect of combined *H. pylori* and BMI status on diabetic status among the study population.

Combined *H. pylori* and BMI status	Diabetic patients n = 93	Non-diabetic patients n = 112	OR (95% CI) *p* value
***H pylori –ve***/**BMI < 25 versus H pylori + ve**/**BMI ≥ 25**			
*H pylori* + ve/BMI ≥ 25	52 (55.91%)	28 (25%)	12.63 (4.440–35.92) < 0.0001*
*H pylori* –ve/BMI < 25	5 (5.37%)	34 (30.35%)	
***H pylori –ve***/**BMI < 25 versus H pylori **−**ve**/**BMI ≥ 25**			
*H pylori* −ve/BMI ≥ 25	20 (21.50%)	13 (11.60%)	10.46 (3.246–33.72) 0.001*
*H pylori* –ve/BMI < 25	5 (5.37%)	34 (30.35%)	
***H pylori –ve***/**BMI < 25 versus H pylori + ve**/**BMI < 25**			
*H pylori* + ve/BMI < 25	16 (17.20%)	37 (33.03%)	2.941 (0.9718–8.897) 0.0773
*H pylori* –ve/BMI < 25	5 (5.37%)	34 (30.35%)	

+ ve: positive, *–*ve: negative, BMI: Body mass index, *H pylori* –ve/BMI < 25 kg/m^2^ group was taken as reference.

*Significant.

### HbA1c blood level in relation to BMI and *H. pylori* status among the dyspeptic sample population

The relationship between *H. pylori* infection and HbA1c level, between BMI and HbA1c level among the study population is illustrated in Table 5. From this table, infected participants had significantly higher mean HbA1c levels compared to those who were uninfected (t = 2.395, *p* = 0.0175). The mean level of blood HbA1c in positive *H. pylori* cases was 7.483 ± 0.2133 vs 6.669 ± 0.2410% in negative cases.

**Table 5:** Comparison of mean HbA1c level of dyspeptic patients as regard *Helicobacter pylori*, BMI status and different combination of BMI and *H. pylori* status.

Variable	Mean HbA1c value (%)	t value	*p* value
***H pylori status***			
*H pylori* + ve	7.483 ± 0.2133	2.395	0.0175*
*H pylori* −ve	6.669 ± 0.2410		
**BMI ≥ 25 kg/m**^2^			
Yes	8.016 ± 0.2356	5.795	< 0.0001*
Non	6.244 ± 0.1840		
**Combined H pylori and BMI status**			
*H pylori* –ve/BMI < 25 kg/m^2^ versus *H pylori* + ve/BMI ≥ 25 kg/m^2^			
* H pylori* –ve/BMI < 25 kg/m^2^	5.805 ± 0.2156	5.444	< 0.0001*
* H pylori* + ve/BMI ≥ 25 kg/m^2^	8.177 ± 0.2881		
*H pylori* –ve/BMI < 25 kg/m^2^ versus *H pylori* −ve/BMI ≥ 25 kg/m^2^			
* H pylori* –ve/BMI < 25 kg/m^2^	5.805 ± 0.2156	4.190	< 0.0001*
* H pylori* −ve/BMI ≥ 25 kg/m^2^	7.625 ± 0.4015		
*H pylori* –ve/BMI < 25 kg/m^2^ versus *H pylori* + ve/BMI < 25 kg/m^2^			
* H pylori* –ve/BMI < 25 kg/m^2^	5.805 ± 0.2156	2.024	0.0459*
* H pylori* + ve/BMI < 25 kg/m^2^	6.550 ± 0.2677		

Mean ± SD, SD: Standard deviation, + ve: positive, *–*ve: negative, BMI: Body mass index, *significant. Comparison using Unpaired t-test. As combination of variable is concerned, HbA1c mean value of *H pylori* –ve/BMI < 25 kg/m^2^ group was taken as reference.

Similarly, our data showed a significantly higher mean of HbA1c level among overweight participants (8.016 ± 0.2356%), than those with normal weight (6.244 ± 0.1840%), (t = 5.795; *p* < 0.0001).

A significant increase in blood HbA1c level was observed when comparing individual combined category of BMI and *H. pylori* status to the reference combined group (*H. pylori* negative and BMI ˂ 25 kg/m^2^), with the peak of blood HbA1c level (8.177 ± 0.2881) in the group having both *H. pylori* positive and BMI ≥ 25 kg/m^2^ vs 5.805 ± 0.2156% in the said reference group (Table 5).

## Discussion

The level of glycated hemoglobin (HbA1c) which results from the non-enzymatic glycosylation of hemoglobin and reflect the integrated blood glucose level for the past 3–4 months^31–33^, was used to assess the diabetic status of our dyspeptic sample population. Among the 205 participants with dyspepsia symptoms enrolled, 93 were diabetics, given an overall diabetes mellitus prevalence of 45.36%. This prevalence seems to be real despite the low prevalence of diabetes reporting in Cameroon (10%) as it is known that, in Sub-Saharan Africa, for every diagnosed person with diabetes, there are one to three undiagnosed cases independently of their dyspeptic status. For instance, the prevalence of undiagnosed diabetes is 60 percent of those with diabetes in Cameroon^34^, 70 percent in Ghana^35^, and over 80 percent in the recent study in Tanzania^36^. However, the present prevalence of diabetes in dyspeptic patients seems to be very high compared to 4% recorded by Smith et al. in their dyspeptic sample population^37^. The recruitment process of diabetic patients in the studied population between the present and this previous studies, may explain such observation. In fact, in their sampling processes, Smith et al. included only dyspeptic patients who were diagnosed previously with T2DM or were previously aware of their diabetes status whereas in our study, all the eligible participant whether they were aware or not of their diabetic status were diagnosed for T2DM. Thus, the incidence rate of diabetic in their study dyspeptic population would be more or less lower than that in our population since in Sub-Saharan Africa, there more undiagnosed diabetes cases for every diagnosed ones. As illustration, out of the 392 eligible subjects in this study, 17 (4.34%: 17/392) were previously aware of their diabetes status (Fig. 1). Age of eligible participants, setting area and study’ community could also explain such observation, since marked discrepancies between the prevalence of diabetes among different communities in Sub-Saharan Africa, independently for dyspeptic status of the population has been reported. The studies from Tanzania^36^, shown an urban-to-rural ratio of five to one, and a ratio of two to one from Cameroon^34^, both confirming the urban–rural discrepancy in diabetes prevalence.

When considering the age of our participants and the obtained diabetes mellitus prevalence, we noticed that as the age increase, the risk to develop diabetes mellitus increase and reach a peak at 55–65 years old (Table 1). This result is in accord with data of the literature which reports that old people are more affected by DMT2 than young ones independently of their dyspeptic status. According to Centers of Diseases Control and Prevention (CDC), in 2008 to 2009, 0.8 per 100,000 new case of DMT2 were detected among children less than 10 years old compared to 11 per 100,000 among those between 10 to 19 years. From the same source, this rate in the said period was approximately 12.3% among adults aged 20 years or older and reach 26.9% among those older than 65 years. Some studies estimate that above 80 years of age there is a 30% probability of having a fully normal glucose metabolism, this figure suggests that the disordered glucose metabolism may be part of a normal ageing process rather than a disease^38^.

The prevalence of diabetes was approximately similar in dyspeptic men and women (48.03 women vs 41.02% men) in this study (Table 1). Currently, men have a higher risk of developing DMT2 than women, due to the fact that they have a more central adipose tissue distribution compared to women.

Participants with low income were significantly less affected by diabetes than their corresponding socioeconomic group (*p* = 0.0103) (Table 2). This may be attributed to the cumulative effects over years of the dietary habit, decrease in physical activity because they have transport facilities, and psychological stress. In fact, people with Middle and high income level in Africa have a dietary habits involving an increase in the consumption of refined sugars and saturated fat through excessive meat consumption and a reduction in fiber intake^39,40^. This dietary habit and physical inactivity have recently reported as potential risk factors for obesity and diabetes mellitus. Our finding is in accordance with previous reports revealing that the prevalence of diabetes in Africa is not uniformly distributed with apparent increases with economic development. It ranged from 4.4% in low-income countries to 5.0% in lower-middle income and 7.0% in the upper-middle income countries^39,40^.

Our data showed that dyspeptic individuals prone to develop diabetes were those with high BMI. Previous reports show that among those suffering from DMT2, 80% are obese and that as the BMI increases, the risk of developing diabetes also increases. For a BMI greater than 35 kg/m^2^, there is an 80 fold greater risk of developing DMT2 over a ten year period than those whose BMI is less than 22 kg/m^2^^41^. There are several facts that helps to understand the implication of increase susceptibility of diabetes in overweight individuals. Firstly, through lipolysis high quantities of non-esterified fatty acid is produced by visceral fat, which leads to an improvement of gluconeogenesis in the liver and an impairment of glucose intake in the muscles^42–44^. Secondly, triglycerides deposits within beta cells induce by high level of non-esterified fatty acids may also interfere with insulin production^42–44^. Also, cytokines like resistin, IL-6 and TNF-α, produced by adipose tissues have been demonstrated to affect insulin effect^45,46^. In fact, previous studies have reported that the expression of GLUT-4, a transporter of glucose is decreased by TNF- α. It is also proved that this cytokines interfere with tyrosine kinase activity at the insulin receptor^45,46^.

Approximately sixty five percent (64.87%) of participants in our sample population were *Helicobacter pylori* seropositive. Our data showed that patients from diabetic group were significantly more *H. pylori* infected compared to those from the non-diabetic group (Table 1). For DM dyspeptic patients, *H. pylori* seropositivity was found to be 73.11% and for the dyspeptic control group it was 58.05% (*p* = 0.0279), suggesting a positive relationship between *H. pylori* seropositivity and diabetes. Our findings goes in the same line with that of Gentiles et al. who reported *H. pylori* infection rate of 74.4% against 50% in their diabetic and control population respectively^7^. It is also in accordance with a report from Hungary suggested that there is an increase in the prevalence of *H. pylori* infection among diabetes mellitus patients^6^ and later supported with studies from developing and developed countries^7–9^. Similarly, Candelli et al. in their study reported that among patients with diabetes, the prevalence of *H. pylori* infection was higher than in the control group, and that three years after a standard eradication treatment, the reinfection rate in the patients with diabetes was higher than in the control group^47^. In fact, it has been shown that *H. pylori* infection increase the rate of the metabolic syndrome in a study conducted on the Japanese population^48^. In the same way, a significant increase in insulin resistance in *H. pylori* infected asymptomatic patients has been shown^49^.

The positive relationship between *H. pylori* seropositivity and diabetes mellitus was still true even when we consider the age and income level of our sample population (Table 3). Our data showed that age and socioeconomic status of participants were also related to diabetes mellitus among *H. pylori* infected patients with a higher risk to develop diabetes among infected participants from ≥ 55 years age group (*p* = 0.0036) and those with high income (*p* = 0.0196). Candelli et al. in their study also found that age and socioeconomic status were related to *H. pylori* reinfection in diabetic patients^47^, but in contrast, they found that people with low income rather than high income were associated to higher incidence of *H. pylori* infection in diabetic patients. However in this study, higher prevalence of *H. pylori* infection was also found among participants with low income level independently to their diabetic status.

Infected patients with high BMI were more prone to develops diabetic mellitus compared with infected patients with normal BMI (*p* = 0.0034) (Table 2). Also, participant with high BMI were more prone to develops diabetic mellitus whether they were infected or not. On the other hands, patients having both *H. pylori* + ve and BMI ≥ 25 kg/m^2^ were significantly more affected by diabetic mellitus than those in the others groups (*p* < 0.0001) (Table 1). Such observations suggest that high BMI and *H. pylori* infection together or not are factors that favor diabetes mellitus development.

Regarding blood HbA1c levels, we noticed a significantly higher mean HbA1c levels (*p* < 0.0001) among participants with high BMI than those with normal body weight (Table 5). A similar result was obtained among infected participants compared with non-infected ones (*p* = 0.0175). This indicates that separately, *H. pylori* infection and high BMI enhance the blood HbA1c level. As *H. pylori* infection increase HbA1c blood levels, one may expect that the incidence rate of high level of HbA1c would be higher in *H. pylori* endemic area. Published data on the prevalence of *H. pylori* infection among patients with gastrointestinal discomfort in Cameroon revealed that because of poverty, malnutrition, poor hygiene and unaffordable heath care, around 64.34–92.2% were infected^17,20^, therefore it is not unexpected to find such a high rate of high HbA1c level in our dyspeptic sample population. Our finding corroborate with that of previous studies on the relationship between HbA1c blood level and *H. pylori* /BMI status. A study performed by Chen and Blaser showed that high BMI and *H. pylori* presence are separately linked with high level of HbA1c and this same study also showed the joint effect of BMI and *H. pylori* infection on the increased level of HbA1c^10^. Similarly, some results showing relevant relationships between chronic *H. pylori* infection and increased level of HbA1c, lowered insulin production in Taiwan were reported^11^. The presence of *H. pylori* in the stomach influences the production of ghrelin and leptin by endocrine cells of the gastric mucosa^50,51^. These two gastric hormones act in metabolic homeostasis through the regulation of food intake and energy consumption. This mechanism could help to understand the relationship between *H. pylori* infection and Hb1Ac levels. Another suggested mechanism could be the presence of cytotoxin associated gene protein, a virulent factor produce by *H. pylori*, which may contribute in the inflammatory disturbance observed in the metabolic syndrome^52^. In addition, *H. pylori* infection results to the up-regulation of various cytokines like Interleukin (IL)-1β, Tumour Necrosis Factor (TNF) and C-reactive protein (CRP). These cytokines may interfere in the pancreatic β cells function and on insulin effect^45,46^. More proofs showing the role of TNF-α and IL-1 beta on β cells dysfunction such as β cells apoptosis and slowing down of insulin production has been documented^45,46^.

Contrarily, no link was found between *H. pylori* and diabetes in some studies^14,15,53,54^. For example, Xia et al. in their study found not significant difference of *H. pylori* seropositivity between diabetic and non-diabetic subjects^14^. Oluyemi et al. in Nigeria^55^ and others studies form various regions in the world such as Romania^53^, Italy^56^, China^57^ and Turkey^58^ obtained similar results. The differences in the reports regarding the relationship between *H. pylori* and diabetes may be linked to the genetic diversity of *H. pylori* strains. Significant geographic genetic variation in specific virulence genes (cytotoxin-associated gene product (CagA), the vacuolating toxin (VacA) and the adhesion protein BabA2) that contribute to the variable risk of diverse clinical outcomes of *H. pylori* infection has been demonstrated^59–61^. Since, these virulence factors participate in the pathogenic process of *H. pylori* infection in the stomach, the geographic diversity of *H. pylori* virulence factors may explain the variation in the strength of the relation between diabetes and *H. pylori* infection. However, this hypothesis needs to be evaluated.

## Conclusion

This study found that the prevalence of *H. pylori* infection among diabetic patients was significantly higher than in non-diabetic patients. Regarding blood HbA1c levels, a significantly higher mean HbA1c levels was noticed among participants with high BMI and among infected participants compared with their corresponding control groups. An increase in intensity of HbA1c blood level was obtained when having both high BMI and *H. pylori* infection. This indicates that separately or together, *H. pylori* infection and high BMI are risk factors for diabetes mellitus. A further attention for the effect of anti *H. pylori* treatment drugs administration in chronic gastritis infections on the evolution of blood HbAc1 level is necessary.

## Data Availability

The datasets used and/or analyzed during the current study are available from the corresponding author on reasonable request.
